# Climate change and *Plasmodium vivax* Malaria Risk in Brazil: Developing adaptive tool for Brazilian Municipalities

**DOI:** 10.1371/journal.pntd.0014298

**Published:** 2026-05-26

**Authors:** Tatiane C. M. Sousa, Sandra S. Hacon, George U. Pedra, Cassia M. G. Lemos, Maria Anice M. Sallum, Simone Ladeia-Andrade, Fabiane B. Reis, Gustavo Arcoverde, Lincoln Alves, Jean Ometto

**Affiliations:** 1 Department of Epidemiology, Institute of Social Medicine, University of State of the Rio de Janeiro, Rio de Janeiro, Brazil; 2 National School of Public Health, Oswaldo Cruz Foundation (Fiocruz), Rio de Janeiro, Brazil; 3 Brazilian Institute of Space Research (INPE), São José dos Campos, ‌‌São Paulo, Brazil; 4 Departamento de Epidemiologia, Faculdade de Saúde Pública, Universidade de São Paulo, São Paulo, Brazil; 5 Laboratory of Parasitic Diseases, Oswaldo Cruz Institute, Oswaldo Cruz Foundation (Fiocruz), Rio de Janeiro, Brazil; Gdanski Uniwersytet Medyczny, POLAND

## Abstract

Climate change impacts ecosystems and health sectors, increasing the incidence of climate-sensitive diseases like malaria, mainly in tropical countries. This study assesses malaria risk, particularly related to *Plasmodium vivax*, under climate change scenarios for 2030 and 2050 for two greenhouse gas emission scenarios (RCP 4.5 and RCP 8.5), using the AdaptaBrasil MCTI approach, which supports decision-makers in enhancing climate adaptation strategies. A multilevel analysis was employed to identify key climate variables influencing malaria incidence (temperature, relative humidity, and the Simple Daily Intensity Index (SDII)). A logistic Binary Model was applied to estimate the climate threat associated with malaria incidence in all Brazilian municipalities for baseline and future scenarios. The Vulnerability Index, comprising Sensitivity and Adaptive Capacity dimensions, highlighted social susceptibility and healthcare access as crucial factors driving higher vulnerability. Road networks and land use factors shaped the Exposure Index, and the Climate Threat Index was based on maximum temperature, SDII, and relative humidity. Under both scenarios, the findings show a growing malaria risk across Brazil, with the most significant impact in the Amazon, expanding to the other regions, mainly the Southeast and Northeast, by 2050. Maximum temperature increases (β = 0.35) emerged as the most influential factor, followed by SDII (β = 0.19) and relative humidity (β = 0.12). These results emphasize the need for targeted public health and environmental interventions to address rising malaria risks, particularly in the Amazon. This study offers critical insights into the relationship between climate change and malaria, informing future policies for climate adaptation and public health preparedness in Brazil.

## Author summary

Climate change is expected to alter the distribution and transmission of malaria, particularly in regions where environmental conditions strongly influence mosquito populations and parasite development. In Brazil, *Plasmodium vivax* is the most widespread malaria parasite, and understanding how climate variability affects its transmission is essential for public health planning. In this study, we analyzed the malaria risk under current and future climate conditions across Brazilian municipalities. By integrating climate data, epidemiological information, and indicators of vulnerability, exposure, and adaptive capacity, the tool identifies areas where changing climatic patterns may increase or decrease transmission risk. Our results highlight that some regions may face higher risk due to warming trends and environmental suitability, while others may experience reduced transmission. The tool, which includes all obtained results (AdaptaBrasil MCTI), is designed to support decision-makers by providing accessible, location-specific information to guide prevention and control strategies. By anticipating potential changes in malaria risk, this approach can help strengthen climate-resilient health systems and improve preparedness at the local level, including efforts for malaria elimination.

## Introduction

Climate change alters the water cycle and affects the frequency and intensity of extreme events, posing substantial risks to ecosystems and various sectors of society, including healthcare, and the occurrence of climate-sensitive diseases like malaria [1,2].

Malaria remains a significant health concern, substantially impacting populations in various parts of the world, particularly Central and Western Sub-Saharan Africa. In 2023, the estimated number of cases in the world was 263 million, an increase of 11 million cases from 2022. Regarding mortality, despite a reduction in malaria-related deaths from 2000 to 2019 worldwide (from 861,000 –567,000), there was an upswing in fatalities in 2020, with 622,000 deaths, followed by a reduction, achieving 597,000 deaths in 2023; the number is still above the number of deaths in 2019. This increase is attributed to the impact of the COVID-19 pandemic on malaria health services between 2020 and 2022 [3].

In the Americas, a reduction in malaria cases and deaths was also recorded between 2000 and 2023, with a decrease from approximately 1.6 million cases to 0.55 million (65.4%) and from 896 to 342 deaths (61.8%). The number of malaria cases in the Americas is still highly concentrated in Amazonian countries, with 76.8% of cases in the continent being recorded in Brazil, Venezuela, and Colombia [3].

In Brazil, reported cases declined in the early 2010s, primarily between 2012 and 2016, with a reduction from 242,756 cases in 2012–129,242 cases in 2016. In 2017, there was an increase in cases, with 194,425 cases recorded that year. Between 2018 and 2024, the number of cases decreased from 194,572–142,006, but was still higher than that recorded in 2016 [4]. Cases in Brazil are highly concentrated in the Amazon region, with 97.6% of the cases registered in the country in 2024 originating from this region [5].

Approximately 41% of municipalities in the Brazilian Amazon, the main region with autochthonous transmission cases, were considered to have achieved elimination, while 49.3% were classified as in the process of elimination [6]. However, in 2015, an increase in malaria cases was recorded in the Amazon region, involving both *Plasmodium vivax* and *Plasmodium falciparum* [7]. The rise in *P. falciparum* cases is particularly concerning as it is associated with more severe disease cases and deaths [8].

The distribution in the Amazon region is heterogeneous, with areas varying from high transmission to disease-free [9]. The elevated incidence of malaria in the Amazon is linked to various environmental, socioeconomic, and climatic factors, which create conditions conducive to the proliferation of malaria vectors in the region [10]. In addition to the Amazon region, malaria cases have also been reported in the Atlantic Forest biome, although with a much lower burden than in the Amazon. In 2022, 66 municipalities outside the Amazon region reported at least one locally acquired case of the disease, representing 1.4% of the municipalities with locally acquired cases in the country throughout the entire year [11].

According to the Ministry of Health, although the incidence of malaria is low in the extra-Amazonian region, residual transmission persists in areas of the Atlantic Forest biome, including the states of São Paulo, Minas Gerais, and Rio de Janeiro, among others [12]. Between 2011 and 2018, the number of malaria cases with a probable site of infection in these states increased by 360%, from 52 to 239 cases. Of the 239 cases reported in 2018, 137 were caused by *Plasmodium falciparum*. In the subsequent years, the number of cases declined, reaching just 12 in 2024, including 10 cases of *P. vivax* and 2 of *P. falciparum* [13].

The WHO Global Technical Strategy for Malaria aims to reduce cases in all affected countries by at least 90% by 2030 and eliminate malaria in 35 countries by the same year [14]. In Brazil, the objective is to eliminate malaria gradually by 2035, with several challenges [15]. Although there has been a downward trend in overall malaria infections since late 2017, cases caused by *Plasmodium falciparum* have steadily increased since 2015. Additionally, the rising incidence of malaria among Indigenous populations and in gold mining areas presents further obstacles to the country’s elimination efforts [16].

The expansion of malaria cases in the Brazilian Amazon in 2016 can be attributed to different factors, primarily associated with human economic activities in the region, such as gold mining, in conjunction with the ecological conditions of the forest and/or climatic conditions in the region. Climate variables such as humidity, temperature, and rainfall affect vector ecology, including vector lifespan, probability of egg survival, and development time from eggs to adults [17,18]. Factors such as expanding agriculture and livestock areas, deforestation, and mining have increased environments favorable to the disease vector [6,19]. Demographic characteristics, including migratory processes, also contribute to the intensification of disease incidence and case expansion [20]. The importation of cases due to population migration has been identified as one of the factors hindering malaria elimination in certain municipalities in the Amazon, primarily due to the proportionally higher movement of cases between states in the region [21]. Since 2018, the rise in illegal mining activities, known regionally as “garimpos,” have contributed to the increase in malaria cases in the Brazilian Amazon. The *garimpo* promotes intense and extensive changes in the environment, such as deforestation, destruction of riparian zones and riverbeds, unstructured occupation, and migratory processes involving labor displacement without monitoring and health surveillance, particularly in Indigenous territories [22].

Besides the environmental and socioeconomic factors, malaria has threatened human health, especially in countries conducive to its expansion, because of climate change [23]. Like other vector-borne diseases, climatic factors strongly influence malaria vector occurrence, particularly variables such as precipitation, temperature, humidity, and the occurrence of El Niño [24]. While many models have identified the increase in malaria cases in different climate change scenarios, surveillance and adaptation efforts for this public health emergency have not been sufficient to ensure an effective response at several governance levels. The recent surge in cases in various regions, such as Brazil, underscores that projections are becoming a reality [25].

Considering the diversity and complexity of drivers associated with malaria occurrence in Brazil, controlling the disease and its vectors depends on measures involving various decision-makers in different sectors and levels. For instance, the health sector promotes epidemiological surveillance and assists infected individuals. Simultaneously, environmental agencies are tasked with monitoring and surveilling ecological and climatic parameters, among other activities. Therefore, tools for surveillance and response to the increased incidence of malaria in different climatic scenarios should include a variety of indicators to facilitate the decision-making process for various stakeholders. Given that *Plasmodium vivax* accounts for the majority of malaria cases in Brazil, understanding its climate sensitivity is critical for informing adaptation and elimination strategies.

This study aims to assess the potential impacts of climate change on malaria transmission risk with emphasis on *P. vivax*, occurrence, and geographic distribution in Brazil, regarding the climate change scenarios for 2030 and 2050 for two greenhouse gas emission scenarios (RCP 4.5 and RCP 8.5). To achieve this, we identified the different indicators associated with malaria occurrence in Brazil and considered climate projections for global warming scenarios using Representative Concentration Pathways (RCP) 4.5 and 8.5. These results were developed using the AdaptaBrasil MCTI approach, an open-access platform to evaluate malaria incidence risk in different projections in different climate change scenarios in 5,570 Brazilian municipalities.

### AdaptaBrasil MCTI Approach

The AdaptaBrasil MCTI platform, AdaptaBrasil, has been developed since 2015. It aims to consolidate, integrate, and disseminate information to advance the analysis of climate change impacts in Brazil [26]. This information supports institutional authorities in the public and private sectors in providing adaptation actions across various strategic sectors, such as water, food, and energy security. Anticipating the locations in which future outbreaks are most likely to occur allows scarce surveillance resources to be deployed effectively.

The indicators are based on the risk concepts from the Intergovernmental Panel on Climate Change (IPCC), where climate-related risks result from the interaction between climate threats (hazardous events and trends) and the vulnerability and exposure of human and natural systems, including their adaptive capacity [27].

The indicators are organized by levels and dimensions. Level 5 represents simple indicators (without aggregation), while Level 4 comprises thematic indicators that aggregate simple ones. Level 3 corresponds to sensitivity and adaptive capacity indexes, and Level 2 includes vulnerability, exposure, and threat indexes. Threat indexes are estimated for a Baseline period and projected for two IPCC climate scenarios (RCP 4.5 and 8.5) and two time periods (2030 and 2050), resulting in four scenarios. Level 1 aggregates vulnerability, exposure, and threat indexes to form the malaria risk index under different climate change scenarios.

The vulnerability and exposure indicators are not necessarily expected to be directly influenced by climate change. Instead, they are associated with malaria occurrence and transmission in Brazil through different pathways, such as the creation of environments conducive to the development of the mosquito vector, the introduction or spread of the etiological agents, or the absence of effective surveillance systems to control the disease and support affected individuals.

### Climate model and approach

Brazil is one of the most vulnerable regions to extreme climate events, especially in recent decades, where these events threaten the socio-ecological system, health, infrastructure, and economy. Our work simulates regional climate variability over Brazil during a baseline period (1986–2005) and under two greenhouse gas emission scenarios for two future periods. The emission scenarios are the intermediate RCP 4.5 and the extreme RCP 8.5, and the future periods are 2021–2040 and 2041–2060, centered around the years 2030 and 2050, respectively [16]. These time slices are consistent with the framework adopted by the IPCC AR5/CMIP5, and they are also those applied in the Brazilian national adaptation platform AdaptaBrasil MCTI. Furthermore, recent regional application of CORDEX-CORE simulations for Tropical Brazil explicitly employs the same temporal segmentation [28]. While other authors evaluated the mild (RCP2.6) and extreme (RCP8.5) scenarios, in this study, we selected the intermediate (RCP4.5) and extreme (RCP8.5) pathways. This choice was made because the mild scenario (RCP2.6) is considered less realistic, given that several climate thresholds have already been surpassed, and therefore was not relevant for this initial assessment. Using RCP4.5 alongside RCP8.5 provides a more meaningful contrast between intermediate and high-emission trajectories, which are more consistent with the objectives of our analysis.

These data are utilized to calculate different climate-related threats for various sectors under the AdaptaBrasil Project, which aims to provide tools and scenarios of climate change for the future, which can be used for planning, considering local and regional resilience capacity, allowing managers to apply technologies that may minimize and control the impacts of climate emergency in Brazil.

## Methods

**Fig 1:**
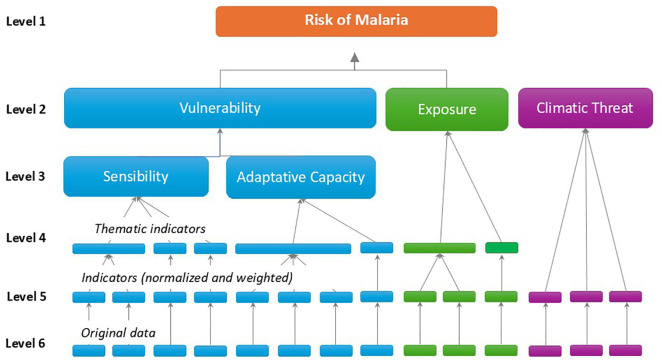
Hierarchical Levels of the Indicators and Index Composition in the AdaptaBrasil Approach. Level 1 represents the estimated risk of malaria occurrence in each municipality. Level 2 comprises the estimated vulnerability, exposure, and climate threat indices. Level 3 refers to sensitivity and adaptive capacity, forming the vulnerability index. Level 4 consists of thematic indicators, which are composed of one or more simple indicators. These simple indicators were normalized and weighted by experts to Brazilian biomes at Level 5. Finally, Level 6 corresponds to the original data for estimating each indicator.

We assessed the incidence and spatial distribution of malaria in Brazil and proceeded with two main steps. First, we identified socio-ecological indicators associated with malaria incidence, constituting the dimensions of vulnerability and exposure (Fig 1). Subsequently, we identified climatic variables linked to malaria cases and estimated climatic threats, considering the specificities of the biome.

In Fig 1, each dimension is represented by a color (vulnerability in blue, exposure in green, and climate threat in purple). Level 6 refers to the raw data, and level 5 corresponds to the transformation of each raw data point into a simple indicator, normalized from 0 to 1 and weighted by biome. Level 4 refers to the indicators, composed of one or more simple indicators, as illustrated by the larger or smaller rectangle at this level. It should be noted that no thematic indicators were used in the composition of the climate threat, as it was composed exclusively of simple indicators. Level 3 applies exclusively to the vulnerability dimension, since this is composed of the sensitivity and adaptive capacity indices, which in turn are composed of thematic indicators. In the end, the index for each dimension (vulnerability, exposure, and climate threat) comprises the malaria risk.

### Data sources

The socio-ecological indicators were selected and defined through workshops with consultation groups with experts in malaria incidence and behavior concerning the climate variables and spread in Brazil. The expert elicitation process was performed with experts in malaria, environmental health, and climate change who participated in four meetings between October 2020 and April 2021. In these sessions, we presented the project methodology, and five experts recommended indicators associated with each dimension of the AdaptaBrasil methodological approach. Additionally, the experts provided weightings for each indicator based on the Brazilian biomes (Amazon, Caatinga, Cerrado, Atlantic Forest, Pantanal, and Pampa). A detailed description of the simple indicators and the weight by biome is provided in S1 and S2 Tables.

Demographic and socioeconomic data were obtained through the 2010 census by the Brazilian Institute of Geography and Statistics (IBGE) [29]. Environmental data were sourced from the MapBiomas platform [20]. Data on malaria cases and deaths were obtained from the Painel Malaria, maintained by the Ministry of Health [4].

The climate data were derived from the Coordinated Regional Climate Downscaling Experiment (CORDEX) project, which provides high-resolution climate projections through regional downscaling techniques [30]. CORDEX is an initiative of the World Climate Research Program (WCRP), and it plays a crucial role in disseminating regional-scale climate data using coordinated Regional Climate Downscaling (RCD) techniques [31], covering diverse continental regions worldwide. The CORDEX project generates climate projections employing a variety of Regional Climate Models (RCMs) across 14 geographical domains, encompassing major continental areas. Its outputs include multivariable time series at various spatial and temporal resolutions, capturing different climate scenarios. The climatic variables analyzed were minimum temperature, maximum temperature, Simple Daily Intensity Index (SDII) of precipitation, and relative humidity.

### Statistical analysis

After obtaining the raw data on socio-ecological indicators, the data underwent outlier treatment using the Winsorization technique. They were normalized between 0 and 1 and subsequently weighted according to the weights specified by malaria experts for each indicator in each Brazilian biome. The median of the values obtained from lower-level indicators was used to estimate composite indicators formed by more than one indicator. The same method was applied when estimating the indices that comprise higher levels in the hierarchical structure presented in Fig 1.

A multilevel analysis model was used to identify the climatic variables associated with the incidence of malaria in Brazil. The dependent variable was the malaria incidence rate per 100,000 inhabitants at the municipal level for each Brazilian municipality between 2001 and 2005. This interval corresponds to the intersection between the climate baseline period (1986–2005) and the available malaria notification data across the Brazilian territory.

The multilevel models were performed separately for each biome where malaria cases were identified in Brazil (Amazon and Atlantic Forest). In each model, one level represented the spatial scale (municipality) and the temporal scale (year). When necessary, a function was applied to account for the excess of zero-case observations.

Based on the analysis of the multilevel models, as presented in the Results section, the climatic variables used in the subsequent modeling stage were the annual mean of maximum temperature (TASMAX), the annual mean of relative humidity, and the annual mean of the SDII.

A Logistic Regression Model with a binomial distribution was used to estimate the climate threat index, considering the average number of cases per Brazilian municipality. This model produced an empirical cumulative distribution function, calculating the probability of malaria occurrence given the number of cases reported in each municipality.

The dependent variable (malaria occurrence) was categorized based on each municipality’s average probability of occurrence, with a value of “0” assigned to municipalities with a probability lower than 40% and a value of “1” assigned to municipalities with a probability equal to or higher than 40%. This categorical variable represented the probability of malaria occurrence in further analyses.

All statistical analyses were conducted using R software version 4.2.0.

## Results

### Socio-ecological Indicators and Index

**Fig 2:**
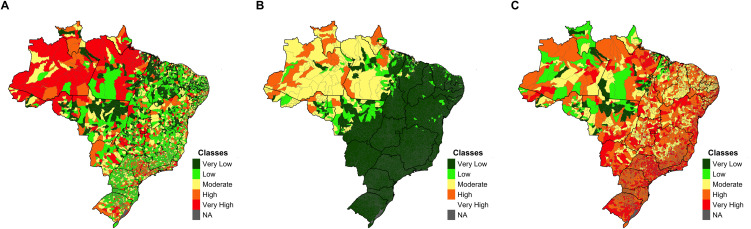
Classification of Brazilian Municipalities by Vulnerability (A), Sensitivity (B), and Adaptive Capacity (C) Indexes (NA = No data available). Results of the vulnerability index estimation (A) for all Brazilian municipalities. The vulnerability index comprises the sensitivity (B) and adaptive capacity (C) indexes. The sensitivity index consists of thematic indicators related to social susceptibility, population mobility, and the epidemiological profile of malaria. The adaptive capacity index is based on the thematic indicator of health services coverage. The results for each index (vulnerability, sensitivity, and adaptive capacity) are classified into five risk levels: very low, low, moderate, high, and very high. The category NA (No Data Available) was used when data were unavailable. Maps were generated by the authors using R software. Municipality and state boundary shapefiles (BR_Municipios_2024; BR_UF_2024) were obtained from the Brazilian Institute of ‌‌Geography and Statistics (IBGE) and are available in the public domain: https://www.ibge.gov.br/geociencias/organizacao-do-territorio/malhas-territoriais/15774-malhas.html.

After the workshops with experts, eight simple socio-ecological indicators were identified to compose the Vulnerability Index (Fig 2A), which comprises the Sensitivity Index and the Adaptive Capacity Index (Table 1, Level 5). These indicators were selected based on their relevance for characterizing malaria vulnerability in Brazil, as identified by experts during the workshops. They reflect key dimensions of sensitivity and adaptive capacity, as outlined in the conceptual model adopted in this study. The thematic indicators for the Sensitivity Index included social susceptibility, population mobility, and the malaria epidemiological profile (Fig 2B). Conversely, the thematic indicator that composed the Adaptive Capacity Index was healthcare services (Fig 2C). Table 1 presents the indicators distributed according to the levels suggested by the hierarchy used to define and analyze the Vulnerability Index. Concerning Adaptive Capacity, the lower the health service access indicator values, the higher the risk. Therefore, the colors are inverted in Fig 2.

**Table 1:** Simple and Thematic Indicators and Indixes: Constructing the Vulnerability Index.

Index (Level 2)	Index (Level 3)	Thematic indicators (Level 4)	Simple indicators (Level 5)
Vulnerability	Sensitivity	Social susceptibility	Lack of access to adequate sanitation
			Municipal Human Development Index
		Population mobility	Municipal tourism
			Recent immigration
		Epidemiological profile of malaria	API - Estimation of the Annual Parasite Index (API) of the municipality
			Occurrence of *P. falciparum* malaria cases
	Adaptative Capacity	Health services coverage	Primary care coverage
			Proportion of notified malaria cases diagnosed on time for treatment outcome.

As observed in Fig 2, the northern region of Brazil, where the Brazilian Amazon is located, concentrates the highest number of municipalities classified as having very high vulnerability, followed by municipalities in the Central-West region (Fig 2A). Similarly, municipalities with a Sensitivity Index classified as medium to high are in the Amazon, while the rest of the country exhibits very low sensitivity (Fig 2B). Contrastingly, the Adaptive Capacity Index was higher in municipalities in the southern portion of the Amazon, primarily due to greater coverage of primary healthcare services in these municipalities (Fig 2C).

### Exposure index

**Fig 3:**
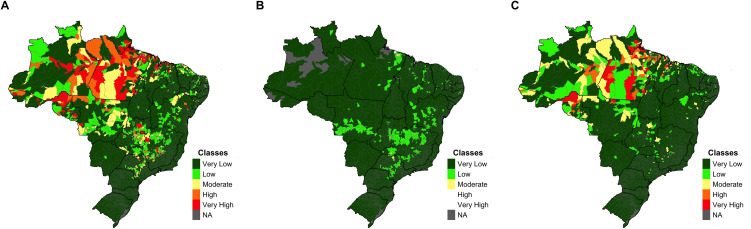
Classification of Brazilian Municipalities by Exposure Index (A) and the Corresponding Thematic Indicators of Road Network (B) and Land Use and Cover (C) (NA = No data available).

The Exposure Index comprised two thematic indicators: the road network and land cover and use. The road network indicator consisted of a single straightforward indicator: road density. The land use and land cover indicator comprised five simple indicators: (i) the presence of rural settlements, (ii) the presence of mining activities, (iii) recent deforestation, (iv) rural population, and (v) water bodies (Table 2). The selection of these five exposure indicators was based on expert consultation; prioritizing factors related to population exposure to favorable conditions for the presence and development of malaria vectors. In this case, there is no index at Level 3, as presented in Table 1, for the Vulnerability Index. The final Exposure Index and its thematic indicators are presented in Fig 3.

**Table 2:** Simple and Thematic Indicators: Constructing the Exposure Index.

Index (Level 2)	Thematic indicators (Level 4)	Simple indicators (Level 5)
Exposure	Road network	Highway density
	Land cover and use	Presence of recent rural settlements
		Presence of mining activities
		Recent deforestation
		Rural population
		Proportion of the water bodies’ coverage

Results of the Exposure index estimation (A) for all Brazilian municipalities. The Exposure index comprises the thematic indicators of Road Network (B) and Land Use and Cover (C). The results for each index are classified into five risk levels: very low, low, moderate, high, and very high. The category NA (No Data Available) was used when data were unavailable. Maps were generated by the authors using R software. Municipality and state boundary shapefiles (BR_Municipios_2024; BR_UF_2024) were obtained from the Brazilian Institute of Geography and Statistics (IBGE) and are available in the public domain: https://www.ibge.gov.br/geociencias/organizacao-do-territorio/malhas-territoriais/15774-malhas.html.

The highest concentration of municipalities classified with very high exposure is also located in the North region of Brazil, followed by the country’s Central-West and Southeast regions (Fig 3A). Considering the road network results, we can see a higher highway density in the municipalities in the Southeast region (Fig 3B). In contrast, the highest ranges of land cover and use changes are concentrated in the North region (Amazon biome) (Fig 3C).

### Climate threat index

Since malaria cases in Brazil are almost exclusively reported in the Amazon and Atlantic Forest biomes, the multilevel analysis focused on these biomes, resulting in two models. The first model considered malaria cases recorded in municipalities within the Amazon biome, while the second model focused on cases in municipalities within the Atlantic Forest biome. In these multilevel models, one level represented the spatial scale (municipality) and the temporal scale (year). A function was applied for the Atlantic Forest biome to account for the excess of zero-case observations in the sample units, as this biome contains the largest number of municipalities with low malaria incidence. The cases used in this analysis covered the years 2001–2005. From the results of both models, it was possible to identify the climatic variables that should be used in estimating the climatic threat for malaria incidence.

The initial composition of the Climate Threat Index was derived from the results of the mixed-effects models, where the climatic variables were positively associated with malaria incidence in both biomes (Amazon and Atlantic Forest). In the Amazon, the climate variable associated with malaria rates was SDII (p < 0.000), while in the Atlantic Forest, these variables were maximum temperature (p = 0.001), SDII (p < 0.000), and relative humidity (p = 0.005) (Table 3). Based on these results, these three climatic variables were selected to estimate the climate threat for malaria incidence in Brazil.

**Table 3:** Results of the Analysis of the Association between Malaria Incidence (2000– 2005) and Climatic Variables Using Multilevel Models for the Amazon and Atlantic Forest Biomes.

	Amazon			Atlantic Forest		
*Coef.*	*St. Error*	*p-value*	*Coef.*	*St. Error*	*p-value*
**Minimum temperature**	851.19	440.77	0.874	-0.173	0.100	0.084
**Maximum temperature**	-295.65	655.46	0.652	0.372	0.116	0.001
SDII*	-2225.87	599.96	0.000	-1.789	0.153	0.000
**Relative humidity**	39.08	85.59	0.054	0.055	0.019	0.005

Note: *SDII = Simple Daily Intensity Index.

**Fig 4:**
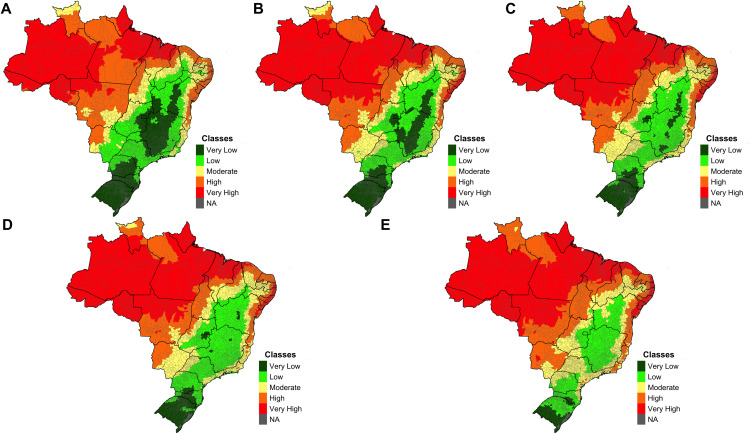
Climate Threat Index for the Baseline, 2030 and 2050 Under the RCP 4.5 e 8.5 scenarios (NA = No data available). Climate risk was estimated for a baseline period (2001–2005) and two future periods (2030: 2021–2040; 2050: 2041–2060) under two emission scenarios (RCP 4.5 and RCP 8.5). Accordingly, five maps are presented: Baseline **(A)**, RCP 4.5 for 2030 **(B)**, RCP 4.5 for 2050 **(C)**, RCP 8.5 for 2030 **(D)**, and RCP 8.5 for 2050 **(E)**. The results for each index are classified into five risk levels: very low, low, moderate, high, and very high. The category NA (No Data Available) was used when data were unavailable. Maps were generated by the authors using R software. Municipality and state boundary shapefiles (BR_Municipios_2024; BR_UF_2024) were obtained from the Brazilian Institute of Geography and Statistics (IBGE) and are available in the public domain: https://www.ibge.gov.br/geociencias/organizacao-do-territorio/malhas-territoriais/15774-malhas.html.

Based on the results from Table 3, the increase in temperature is the factor that most strongly influences the potential occurrence of malaria (β = 0.35), followed by SDII (β = 0.19) and relative humidity (β = 0.12). Fig 4 illustrates the results obtained for climate threats during the baseline period and the estimates for 2030 and 2050 under the RCP 4.5 and 8.5 scenarios.

### Malaria risk index

The median estimates obtained from the final Vulnerability, Exposure, and Climate Risk Indexes were used to calculate the Malaria Risk indicators for Brazilian municipalities. The final estimation indicated each Brazilian municipality’s risk of malaria infection in the Baseline period: 2030 RCP 4.5, 2030 RCP 8.5, 2050 RCP 4.5, and 2050 RCP 8.5.

**Fig 5:**
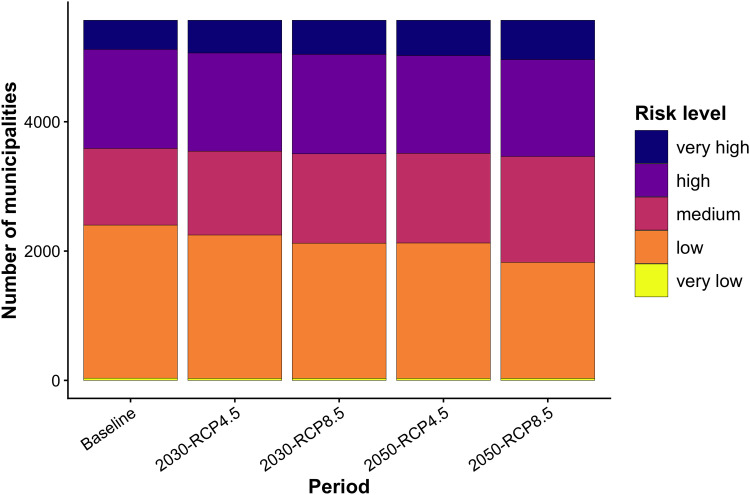
Number of Brazilian Municipalities by Risk Categories During the Baseline, 2030, and 2050 Periods Under RCP 4.5 and 8.5 Scenarios. The bar chart shows the number of municipalities classified into five malaria risk levels (very low, low, medium, high, and very high) for the present and under projected climate scenarios for 2030 and 2050, using RCP 4.5 and RCP 8.5 pathways. A slight increase in the number of municipalities ‌‌classified as high and very high risk is observed in future scenarios, especially under RCP 8.5.

**Fig 6:**
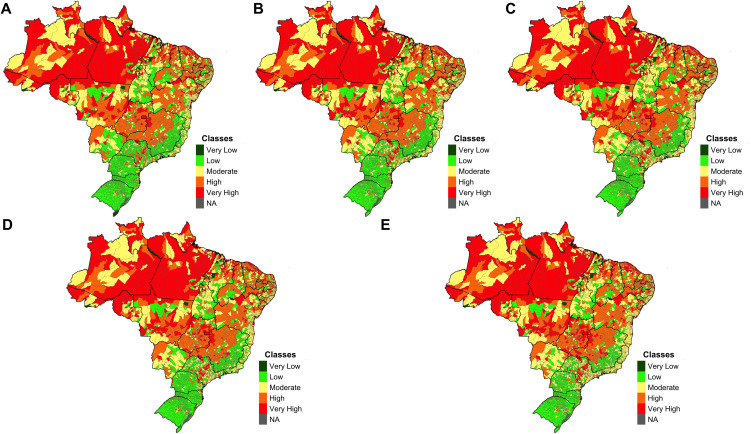
Malaria Risk Index for the Baseline, 2030, and 2050 Periods Under RCP 4.5 e 8.5 Scenarios (NA = No data available). The maps display the classification of all Brazilian municipalities according to malaria risk levels under current conditions (baseline) and future projections for 2030 and 2050, based on RCP 4.5 and RCP 8.5 emission scenarios. Five maps are presented: Baseline **(A)**, RCP 4.5 for 2030 **(B)**, RCP 4.5 for 2050 **(C)**, RCP 8.5 for 2030 **(D)**, and RCP 8.5 for 2050 **(E)**, illustrating potential spatial shifts and areas of increased vulnerability due to climate change. Maps were generated by the authors using R software. Municipality and state boundary shapefiles (BR_Municipios_2024; BR_UF_2024) were obtained from the Brazilian Institute of Geography and Statistics (IBGE) and are available in the public domain: https://www.ibge.gov.br/geociencias/organizacao-do-territorio/malhas-territoriais/15774-malhas.html.

Considering the periods and scenarios analyzed, most Brazilian municipalities present a low or very low risk during the Baseline period (43.12%). In the subsequent periods, there was an increase in the number of municipalities falling into the medium risk category, representing 21.3% during the baseline period and reaching 29.4% in the 2050 RCP 8.5 scenario. Regarding municipalities in the high or very high-risk categories, an increase in municipalities is observed across all periods and scenarios analyzed, rising from 35.6% in the Baseline to 37.8% in the 2050 RCP 8.5 scenario, suggesting we are following the worse climate scenario pathway. This illustrates the growing number of Brazilian municipalities in the medium to very high-risk categories for the occurrence or increase in malaria incidence in Brazil (Fig 5). In all periods and scenarios analyzed, municipalities with high or very high risk are concentrated in the Amazon, followed by central regions of the country. In the RCP 8.5 scenarios for the periods 2030 and 2050, there is a noticeable expansion of municipalities with medium to very high risk in the Northeast and Southeast regions of the country (Fig 6).

A more detailed comparison between the Baseline and the RCP 4.5 scenarios for 2030 and 2050 reveals a gradual increase in malaria risk across Brazilian municipalities. There are few differences, with an increase in the number of municipalities classified as very high risk, particularly in the Northeast and central regions of the country. This increase becomes more evident when observing the expansion of municipalities with high malaria risk in these regions. In general, the same areas identified as having high and very high risk during the baseline period remain unchanged. A similar pattern is observed across both scenarios for the 2050 period, with a more substantial expansion of high-risk areas in the central region of the country, and an increase in municipalities classified as moderate and high risk, many of which previously had lower risk levels, especially along the coastal regions. The increase in areas with a higher risk of disease incidence occurs mainly in the RCP 8.5 scenario, which predicts more intense GHG emissions.

## Discussion

As expected, the highest vulnerability and exposure indicators values, climate threat, and final risk for increased malaria cases refer to municipalities within the Brazilian Amazon, both for the baseline period and future scenarios (RCP’s 4.5 and 8.5). The results obtained for the Baseline period stress the socioeconomic and environmental characteristics of the Amazonian biome, which have directly influenced the occurrence and proliferation of malaria vectors and an increasing number of disease cases in recent years [20].

The results indicate that under scenarios of higher greenhouse gas emissions and high temperatures, especially by 2050, malaria cases could expand into regions outside the Amazon, particularly in the southeast and northeast, where the Atlantic Forest is located. The current occurrence of malaria in the Atlantic Forest is classified as a zoonosis, with non-human reservoirs and competent vectors [32]. The prior presence of these reservoirs and vectors is crucial for the increased risk of malaria in these regions under scenarios of greater climatic favorability for the vector, as illustrated by the results for 2030 and 2050.

Studies on malaria transmission in the Brazilian Amazon show short- and long-term influences of climate variables, in addition to ENSO events, and highlight the high degree of heterogeneity in climate effects in the region and the need for proactive, fine-scale malaria control based on weather forecasting and the development of early warning systems to understand the malaria elimination process. These findings are particularly relevant for *P. vivax,* which is the predominant species in Brazil and whose transmission dynamics are known to be sensitive to climatic factors. As the global climate becomes more extreme, the use of climate data in public health planning will be crucial to achieving malaria elimination, especially in vulnerable regions such as the Amazon [33].

Malaria elimination goals in Brazil may also be affected by changes in disease occurrence associated with climate change, especially in new areas and the intensification of transmission in high-risk regions, such as Indigenous lands and areas of illegal mining; in addition to border regions, where migration and movement of people occur systematically, such as in Guyana, Venezuela, and Bolivia. Furthermore, the increase in tropical deforestation stands out among the simple indicators identified by experts and is associated with challenges in controlling malaria in the Brazilian Amazon [34,35]. Efforts to curb deforestation would not only contribute to reducing greenhouse gas emissions in Brazil but also support malaria control efforts and advance the country’s progress toward disease elimination goals.

The AdaptaBrasil Platform provides managers at various levels with planning and response pathways to mitigate the impacts of climate change. At the municipal, regional, and state levels, managers can identify which vulnerability and exposure indicators impact the municipal risk of malaria and the profile of climatic threats that favor vector occurrence. Based on this identification, managers can enhance current epidemiological and environmental surveillance conditions and socio-ecological indicators, such as deforestation control, present rapid response and alert systems to reduce the likelihood of increased risk under future scenarios.

Additionally, the AdaptaBrasil Platform provides the relative weight of each simple indicator identified in this study for every Brazilian municipality. This information enables local and regional decision-makers to determine the most appropriate public policies to mitigate the occurrence and spread of malaria, based on the indicators that most influence the municipality’s malaria risk level. Beyond supporting local-level interventions, the results also inform federal strategies aimed at both mitigating the effects of climate change in urban areas and strengthening epidemiological surveillance systems for the disease. The results also suggest that greater efforts should be made to reduce greenhouse gas emissions, as the highest number of municipalities classified as being at high or very high risk of malaria occurrence is projected under the RCP 8.5 scenarios, which represent more severe emission pathways.

Among the study’s limitations is the scale of analysis conducted at the municipal level for the entire country. This spatial scale is essential for local public agents to intervene in exposure and vulnerability conditions within their territories using adaptive techniques that better respond to the scenarios of exposure and vulnerability. However, it also limits the data that can be used, as only data available nationwide is utilized, even though some regions may have better data quality. Additionally, the lack of an entomological surveillance program on the disease vectors may be a limitation in identifying and estimating ecological indicators favorable to the occurrence of malaria, as the absence of such data restricts the analysis of the variability of available habitats, considering the development and behavior of the vectors, among other factors. Another limitation of the study relates to the selection of socio-ecological indicators. While based on expert recommendations and nationwide data, some relevant factors may not have been captured due to the socio-ecological diversity across Brazilian cities and biomes. Still, the indicators used are supported by previous literature, reinforcing their relevance for understanding malaria dynamics. Future studies at different spatial scales will be necessary to explore additional potential indicators that may contribute to a more comprehensive understanding of malaria dynamics. Finally, validation efforts of the proposed models, considering the results obtained, are necessary to improve the accuracy of climate change adaptation tools and to better inform the development of more effective public policies.

## Conclusion

in conclusion, our findings indicate that under higher greenhouse gas emission scenarios and rising temperatures, malaria transmission may expand beyond the Amazon region into the Southeast and Northeast of Brazil by 2050, with climate change interacting with environmental processes such as deforestation to reshape malaria dynamics. In this context, the AdaptaBrasil Platform supports evidence-based decision-making by integrating municipal-level vulnerability and exposure indicators with climate threat profiles associated with vector occurrence, thereby informing public policies aimed at mitigating malaria risk at local and regional scales.

## Supporting information

S1 Table Simple, thematic indicators and indexes composing the Vulnerability Index, and the biome weights from expert ponderation. (DOCX)

S2 Table Simple and thematic indicators composing the Exposure Index. (DOCX)

## References

[1] Caminade C, Kovats S, Rocklov J, Tompkins AM, Morse AP, Colón-González FJ, et al. Impact of climate change on global malaria distribution. Proc Natl Acad Sci U S A. 2014;111(9):3286–91. doi: 10.1073/pnas.1302089111 24596427 PMC3948226

[2] Fischer EM, Knutti R. Observed heavy precipitation increase confirms theory and early models. Nature Climate Change. 2016;6(11):986–91.

[3] World Health Organization WHO. World malaria report 2024: addressing inequity in the global malaria response. 2024.

[4] Ministry of Health (Brazil). Malaria - Painel Malaria. https://www.gov.br/saude/pt-br/composicao/svsa/cnie/painel-malaria 2025.

[5] Vitor-Silva S, Siqueira AM, De Souza Sampaio V, Guinovart C, Reyes-Lecca RC, De Melo GC. Declining malaria transmission in rural Amazon: changing epidemiology and challenges to achieve elimination. Malar J. 2016;15(1). 10.1186/s12936-016-1326-2 PMC4863332 27165432

[6] Braz RM, Barcellos C. Análise do processo de eliminação da transmissão da malária na Amazônia brasileira com abordagem espacial da variação da incidência da doença em 2016. Epidemiol Serv Saude. 2018;27(3):e2017253. 10.5123/S1679-49742018000300010 30183869

[7] Ayala MJC, Bastos LS, Villela DAM. On multifactorial drivers for malaria rebound in Brazil: a spatio-temporal analysis. Malar J. 2022;21(1). 10.1186/s12936-021-04037-x PMC8851784 35177095

[8] Zekar L, Sharman T. Plasmodium falciparum malaria. StatPearls Publishing. 2024.

[9] Chaves LSM, Sousa TCM, Penha LCF, Hacon SS. Malaria in the Amazon Basin: how climate change and natural disasters create new challenges for an old disease. Planetary health approaches to understand and control vector-borne diseases. 1st ed. Wageningen: Wageningen Academic. 2023. p. 54–91.

[10] Ministry of Health. Boletim Epidemiológico. Dia da Malaria nas Americas - um panorama da Malaria no Brasil em 2022 e no primeiro semestre de 2023. 2024.

[11] Ministry of the Environment. National climate change adaptation plan (Plano Nacional de Adaptação à Mudança do Clima). 2016. https://www.gov.br/mma/pt-br/assuntos/sustentabilidade-2/PlanoNacionalAdaptacaoMudancaClima.pdf

[12] Ministry of Health B. Malaria na região extra-amazônica. https://www.gov.br/saude/pt-br/assuntos/saude-de-a-a-z/m/malaria/malaria-na-regiao-extra-amazonica 2025.

[13] Ministry of Health B. Dados para cidadão a partir da fonte de dados do Sivep-Malaria, Sinan e E-SUS-VS, para notificações do Brasil de 2007 a 2025. https://www.gov.br/saude/pt-br/assuntos/saude-de-a-a-z/m/malaria/malaria-na-regiao-extra-amazonica 2025.

[14] WHO. World Malaria Report 2021. 2021. https://www.who.int/teams/global-malaria-programme/reports/world-malaria-report-2021

[15] Ministry of Health. Elimina Malária Brasil: Plano Nacional de Eliminação da Malária. 2022.

[16] Garcia KKS, Soremekun S, Abrahão AA, Marchesini PB, Drakeley C, Ramalho WM, et al. Is Brazil reaching malaria elimination? A time series analysis of malaria cases from 2011 to 2023. PLOS Glob Public Health. 2024;4(1):e0002845. doi: 10.1371/journal.pgph.0002845 38295141 PMC10830034

[17] Agyekum TP, Botwe PK, Arko-Mensah J, Issah I, Acquah AA, Hogarh JN. A systematic review of the effects of temperature on Anopheles mosquito development and survival: implications for malaria control in a future warmer climate. Int J Environ Res Public Health. 2021;18(14):7255. 34299706 10.3390/ijerph18147255 PMC8306597

[18] Kaye AR, Obolski U, Sun L, Hart WS, Hurrell JW, Tildesley MJ, et al. The impact of natural climate variability on the global distribution of Aedes aegypti: a mathematical modelling study. Lancet Planet Health. 2024;8(12):e1079–87. doi: 10.1016/S2542-5196(24)00238-9 39674197 PMC7617884

[19] MacDonald AJ, Mordecai EA. Amazon deforestation drives malaria transmission, and malaria burden reduces forest clearing. Proc Natl Acad Sci U S A. 2019;116(44):22212–8. 31611369 10.1073/pnas.1905315116 PMC6825316

[20] Arisco NJ, Peterka C, Castro MC. Spatiotemporal analysis of within-country imported malaria in Brazilian municipalities, 2004–2022. PLOS Global Public Health. 2024;4(7):e0003452. doi: 10.1371/journal.pgph.0003452 PMC11249269 39008438

[21] Castro MC, Singer B. Prioritizing COVID-19 vaccination by age. Proceedings of the National Academy of Sciences. 2021;118(15). 10.1073/pnas.2103700118 PMC8053997 33731429

[22] Kulkarni MA, Duguay C, Ost K. Charting the evidence for climate change impacts on the global spread of malaria and dengue and adaptive responses: a scoping review of reviews. Global Health. 2022;18(1):1. doi: 10.1186/s12992-021-00793-2 34980187 PMC8725488

[23] Sousa TCM, Amancio F, Hacon S de S, Barcellos e C. Climate-sensitive diseases in Brazil and the world: systematic review. Revista Panamericana de Salud Publica/Pan American Journal of Public Health. 2018;42. 10.26633/RPSP.2018.85 PMC6385874 31093113

[24] Samarasekera U. Climate change and malaria: predictions becoming reality. www.thelancet.com 2023. 10.1016/S0140-6736(23)01569-6 37517424

[25] de Pina-Costa A, Brasil P, Di Santi SM, de Araujo MP, Suárez-Mutis MC, Santelli ACF e S, et al. Malaria in Brazil: what happens outside the Amazonian endemic region. Mem Inst Oswaldo Cruz. 2014;109(5):618–33. doi: 10.1590/0074-0276140228 25185003 PMC4156455

[26] Pörtner H, Roberts D, Tignor M, Poloczanska E, Mintenbeck M, Alegría A. Climate Change 2022 – Impacts, Adaptation and Vulnerability. IPCC IP on CC. Cambridge University Press. 2023.

[27] van Vuuren DP, Edmonds J, Kainuma M, Riahi K, Thomson A, Hibbard K, et al. The representative concentration pathways: an overview. Climatic Change. 2011;109(1–2):5–31. doi: 10.1007/s10584-011-0148-z

[28] Alves LM, Firpo MÂF, Bettolli ML, Hasson S ul, Guerron OVC, Añazco AA, et al. Projected changes in the frequency of compound hot and dry events over Tropical Brazil in CORDEX-CORE simulations. Clim Dyn. 2024;62(11):10203–16.

[29] IBGE IB de G e E. Área Territorial - Brasil, Grandes Regiões, Unidades da Federação e Municípios. https://ibge.gov.br/geociencias/organizacao-do-territorio/estrutura-territorial/15761-areas-dos-municipios.html 2022. 2024 February 15.

[30] Jacob D, Petersen J, Eggert B, Alias A, Christensen OB, Bouwer LM, et al. EURO-CORDEX: new high-resolution climate change projections for European impact research. Reg Environ Change. 2013;14(2):563–78. doi: 10.1007/s10113-013-0499-2

[31] Giorgi F, Gutowski WJ. Regional dynamical downscaling and the CORDEX initiative. Annu Rev Environ Resour. 2015;40(1):467–90.

[32] Medeiros-Sousa AR, Laporta GZ, Coutinho RM, Mucci LF, Marrelli MT. A mathematical model for zoonotic transmission of malaria in the Atlantic Forest: Exploring the effects of variations in vector abundance and acrodendrophily. PLoS Negl Trop Dis. 2021;15(2):e0008736. doi: 10.1371/journal.pntd.0008736 33591994 PMC7909691

[33] Arisco NJ, Peterka C, Schwartz J, Castro MC. The impact of weather and extreme events on malaria transmission in the Brazilian Amazon: a case-crossover and population-based study. Lancet Reg Health Am. 2025;49:101189. doi: 10.1016/j.lana.2025.101189 40704022 PMC12284361

[34] Laporta GZ, Ilacqua RC, Bergo ES, Chaves LSM, Rodovalho SR, Moresco GG, et al. Malaria transmission in landscapes with varying deforestation levels and timelines in the Amazon: a longitudinal spatiotemporal study. Sci Rep. 2021;11(1):6477. doi: 10.1038/s41598-021-85890-3 33742028 PMC7979798

[35] Laporta GZ, Valle D, Prist PR, Ilacqua RC, Santos TC, Madeira FP, et al. Intermediate forest cover and malaria risk in an Amazon deforestation frontier. Acta Trop. 2025;269:107757. doi: 10.1016/j.actatropica.2025.107757 40749883

