# Supplementary material for: Climate change and Plasmodium vivax Malaria Risk in Brazil: Developing adaptive tool for Brazilian Municipalities
Source: PLoS Negl Trop Dis. 2026 May 26;20(5):e0014298. doi: 10.1371/journal.pntd.0014298 (PMC13221142; doi:10.1371/journal.pntd.0014298)
Supplement: S1 Table — (DOCX) [file pntd.0014298.s001.docx]

**S1 Table – Simple, thematic indicators and indexes composing the Vulnerability Index, and the biome weights from expert ponderation.**

| *Index*  *(Level 2)* | *Index*  *(Level 3)* | *Thematic Indicators*  *(Level 4)* | *Simple Indicator*  *(Level 5)* | *Definition of Simple Indicator* | *Biome weights based on expert workshops* |
| --- | --- | --- | --- | --- | --- |
| Vulnerability | Sensitivy | Social susceptibility | Lack of access to adequate sanitation - A proportion of households lack adequate sanitation in the municipality. | This indicator is estimated by the percentage of permanent private households in the municipality where a general network does not provide water supply and whose sanitation is not carried out by a sewage collection system or septic tank. A higher proportion of households in these conditions indicates greater municipality sensitivity. | Amazon: 7  Caatinga: 3  Cerrado: 5  Atlantic Forest:2  Pampa: 1  Pantanal: 2 |
|  |  |  | Municipal Human Development Index - Low municipality HDI as an indicator of living conditions. | The HDI allows for assessing vulnerabilities related to educational access, income, and overall living conditions, as it encompasses the longevity of the local population. Lower values of HDI-M indicate greater municipality sensitivity to malaria. | Amazon: 7  Caatinga: 3  Cerrado: 5  Atlantic Forest 2:  Pampa: 1  Pantanal: 2 |
|  |  | Population mobility | Municipal tourism - Tourist visits received in the municipality relative to the municipality's total population. | The intensity of tourism in the municipality, originating from Brazilian tourists, allows us to infer a higher sensitivity of the local population to a possible infection due to the arrival of an infected person. | Amazon: 10  Caatinga: 4  Cerrado: 2  Atlantic Forest: 1  Pampa: 1  Pantanal: 1 |
|  |  |  | Recent immigration - Proportion of residents in the municipality for less than one year. | Migration movements, in general, are associated with an increase in the flow of people from different areas and with different epidemiological profiles. In this sense, the arrival of a considerable number of people from another municipality may indicate recent territorial occupation, where socio-environmental changes in the territory may lead to more vector breeders and expose the local population to the vector. This may also lead to the introduction of infected individuals into a municipality without an endemic record of the outcome. | Amazon: 8  Caatinga: 2  Cerrado: 2  Atlantic Forest:1  Pampa: 1  Pantanal: 1 |
|  |  | Epidemiological profile of malaria | API - Estimation of the Annual Parasite Index (API) of the municipality | The Annual Parasite Index (API) refers to the number of positive malaria tests per thousand inhabitants, according to the probable municipality of infection, in the year under consideration. The API is used as an indicator of the risks of malaria occurrence in a specific population and, in this case, indicates greater population sensitivity to malaria in the municipality. | Amazon: 10  Caatinga: 10  Cerrado: 10  Atlantic Forest: 10  Pampa: 10  Pantanal: 10 |
|  |  |  | Falciparum Malaria - the occurrence of falciparum malaria cases in the municipality results in greater sensitivity of the local population to malaria. | The occurrence of falciparum malaria in the municipality indicates that control measures for the outcome and vector are inadequate, resulting in greater sensitivity of the local population to infection and epidemiological exacerbation of the outcome in the municipality. The indicator's estimate is given by multiplying the highest number of falciparum malaria occurrences between 2017 and 2021 for the city by the weight assigned by experts, considering its relative importance to malaria for the Brazilian biomes. | Amazon: 10  Caatinga: 10  Cerrado: 10  Atlantic Forest: 10  Pampa: 10  Pantanal: 10 |
|  | Adaptative Capacity | Health services coverage | Primary care coverage - percentage of the population coverage by primary care coverage in the municipality | The estimated population coverage in primary care is determined by the percentage of the population covered by family health strategy teams and equivalent traditional primary care teams, which are parameterized by the population estimate. According to the National Primary Care Policy, control, prevention, and response actions to malaria mainly refer to the responsibilities of primary care teams and professionals. Thus, the lower the primary care coverage in the municipality, the lower the municipality's response capacity. | Amazon: 10  Caatinga: 4  Cerrado: 4  Atlantic Forest: 3  Pampa: 3  Pantanal: 3 |
|  |  |  | Access to diagnosis and treatment - Proportion of notified malaria cases diagnosed on time for treatment outcome. | According to the Health Surveillance Actions Qualification Program (PQA-VS), the interval between the onset of malaria symptoms and access to treatment is 48 hours. To estimate this indicator, the proportion of notified malaria cases diagnosed on time for treatment outcome for 2021 is multiplied by the weight assigned by experts, considering its relative importance to malaria for the Brazilian biomes. | Amazon: 10  Caatinga: 10  Cerrado: 10  Atlantic Forest: 10  Pampa: 8  Pantanal: 10 |
