# Supplementary material for: Climate change and Plasmodium vivax Malaria Risk in Brazil: Developing adaptive tool for Brazilian Municipalities
Source: PLoS Negl Trop Dis. 2026 May 26;20(5):e0014298. doi: 10.1371/journal.pntd.0014298 (PMC13221142; doi:10.1371/journal.pntd.0014298)
Supplement: S2 Table — (DOCX) [file pntd.0014298.s002.docx]

**S2 Table – Simple and thematic indicators composing the Exposure Index.**

| *Index*  *(Level 2)* | *Thematic Indicators*  *(Level 4)* | *Simple Indicator*  *(Level 5)* | *Definition of Simple Indicator* | *Biome weights based on expert workshops* |
| --- | --- | --- | --- | --- |
| Exposure | Road network | Highway density | The presence of federal and state highways in the municipality facilitates population flow, in addition to altering land use and land cover in the area. Thus, municipalities with such highways exhibit greater exposure to the occurrence and intensification of malaria cases. Based on this exposure criterion, municipalities were classified according to the municipality's density of federal and state highways (km/km²). The density calculation is given by the ratio of highway segments (km) to the municipality's area (km²), which in turn was weighted by the importance assigned by experts considering its relative significance to malaria across Brazilian biomes. | Amazon: 5  Caatinga: 3  Cerrado: 5  Atlantic Forest: 1  Pampa: 1  Pantanal: 1 |
|  | Land use and cover | Presence of recent rural settlements | The presence of settlements in the municipality induces socio-environmental alterations that facilitate the introduction of Plasmodium in a given region, both through the influx of new residents and local environmental changes that lead to conducive spaces for vector breeding and development, often associated with recent and inadequate urbanization. Based on this exposure criterion, municipalities were categorized in a binary manner. | Amazon: 10  Caatinga: 3  Cerrado: 10  Atlantic Forest: 2  Pampa: 1  Pantanal: 2 |
|  |  | Presence of mining activities | The presence of mining activities, both industrial and artisanal, leads to environmental changes that favor the occurrence of malaria vectors. This increases population flow and, consequently, the movement of infected individuals and the arrival of the etiological agent in municipalities where there was no previous record of the outcome. Based on this exposure criterion, municipalities were classified in a binary manner. | Amazon: 9  Caatinga: 5  Cerrado: 6  Atlantic Forest: 1  Pampa: 1  Pantanal: 2 |
|  |  | Recent deforestation | Recent deforestation promotes the presence of ecotones, i.e., ecological transition environments, where environmental factors related to vegetation, soil exposure, and microclimate are altered. In these areas, changes occur in the availability of larval habitats, resulting in increased vector abundance and malaria risk. Thus, the greater the area recently deforested, the higher the local population's exposure to malaria transmission risk in the municipality. | Amazon: 10  Caatinga: 5  Cerrado: 8  Atlantic Forest: 3  Pampa:1  Pantanal: 3 |
|  |  | Rural population | People living in rural areas may be more exposed to malaria vectors, especially in forested areas such as the Amazon biome. This indicator results from multiplying the percentage of the population residing in rural areas by the weight assigned by experts, considering its relative importance to malaria in Brazilian biomes. A higher percentage of the population living in rural areas indicates more significant exposure to the municipality. | Amazon: 10  Caatinga: 7  Cerrado: 8  Atlantic Forest: 2  Pampa: 1  Pantanal: 2 |
|  |  | Proportion of the water bodies' coverage | Water bodies in the municipality may favor environments conducive to the occurrence and development of malaria vectors. Thus, the greater the percentage of the municipality's area covered by water bodies, the greater the population's exposure to malaria. For the development of this indicator, water body coverage refers to the presence of rivers, lakes, reservoirs, dams, aquaculture areas, and other potential water bodies identified through satellite images. | Amazon: 10  Caatinga: 3  Cerrado: 8  Atlantic Forest: 2  Pampa: 1  Pantanal: 2 |
